# Effects of Lactic Acid Bacteria Fermentation on the Physicochemical Properties, Nutritional Value, and Sensory Characteristics of a White Kidney Bean-Based Beverage

**DOI:** 10.3390/foods15183206

**Published:** 2026-09-10

**Authors:** Yewen Xu, Liping Sun, Ying Gu, Yongliang Zhuang, Hao Liang, Yangyue Ding, Xuejing Fan

**Affiliations:** 1 Faculty of Food Science and Engineering, Kunming University of Science and Technology, Kunming 650500, China; hoshinochi@163.com (Y.X.); lpsun@kmust.edu.cn (L.S.); guying@kust.edu.cn (Y.G.); ylzhuang@kmust.edu.cn (Y.Z.); dingyangyue77@163.com (Y.D.); 2 Wuhan Customs Technology Center, Wuhan 430050, China; lianghao--1984@163.com

**Keywords:** white kidney bean, lactic acid bacteria fermentation, plant-based beverage, nutritional properties, sensory evaluation

## Abstract

The increasing demand for sustainable food systems, high-value resource utilization, and dietary diversification has promoted the development of plant-based beverages. This study aimed to develop a white kidney bean-based beverage (WKBB) through lactic acid bacteria fermentation and investigate the effects of different fermentation strategies on its physicochemical properties, nutritional characteristics, volatile compounds, and sensory quality. WKBB was fermented using *Lactiplantibacillus plantarum*, *Limosilactobacillus fermentum*, and a mixed culture of both strains. The results showed that fermentation significantly modified the quality characteristics of WKBB, including changes in its physicochemical properties and free amino acid composition. Furthermore, fermentation promoted the release of phenolic and flavonoid compounds during simulated gastrointestinal digestion. GC–MS analysis revealed that fermentation altered the volatile composition of WKBB and increased the diversity of volatile compounds, thereby contributing to a more complex flavor profile. Sensory evaluation indicated that fermented samples exhibited improved overall sensory characteristics, with mixed-strain fermentation resulting in a more balanced quality profile. These findings demonstrate that lactic acid bacteria fermentation, particularly mixed-strain fermentation, is a promising and sustainable strategy for the development of a white kidney bean-based beverage and for enhancing the utilization of plant-based resources.

## 1. Introduction

Protein is an indispensable nutrient that plays a crucial role in regulating physiological functions. Reports indicate that animal products account for approximately 58% of the daily per capita protein availability in the European Union [1]. Meanwhile, meat consumption in Europe continues to rise, while the intake of plant-based foods remains at a relatively low level, which may result in an inadequate intake of plant proteins and dietary fiber [2]. Nevertheless, plant-based foods are acknowledged as valuable sources of high-quality proteins [3]. They also provide a diverse array of functional constituents and bioactive compounds, including dietary fiber, vitamins B and E, minerals and dietary polyphenols [4,5]. In recent years, plant-based diets have attracted increasing attention because of their distinctive nutritional properties and potential health-promoting benefits [6]. For instance, minerals and vitamins present in plant-based foods help maintain physiological homeostasis, while bioactive amino acids, such as arginine and cysteine, enhance insulin sensitivity and modulate immune responses [7]. Legumes are important components of plant-based diets due to their high content of protein, carbohydrates, dietary fiber, minerals, and other nutrients [8]. In the Chinese market, legume-derived products are available in several categories, including plant-based meat alternatives, plant protein beverages, plant-based yogurts, and bean-based starch noodles [9,10,11,12]. However, legumes often contain antinutritional factors, such as tannins, saponins, phytic acid, and protease inhibitors, which can reduce nutrient bioavailability [13,14]. Among these, phytic acid (inositol hexaphosphate) is a prominent antinutritional factor. It consists of an inositol ring with six phosphate groups. This structure allows phytic acid to chelate mineral cations and form insoluble phytate complexes. As a result, the bioavailability of minerals such as calcium, magnesium, zinc, copper, and iron is reduced [15,16]. In addition, legumes often exhibit beany, earthy, and grassy off-flavors. These flavor attributes can reduce their sensory acceptability [17,18]. Fermentation is a widely used technique in the food industry that can improve sensory attributes, extend shelf life, enhance nutritional value, and reduce toxic compounds and antinutritional factors in foods [19]. Sun et al. [20] prepared mung bean yogurt using *Lactiplantibacillus plantarum* SF28. The fermented product showed higher levels of free amino acids and greater antioxidant capacity, together with reduced levels of off-flavor compounds and increased levels of aroma compounds, resulting in an improved aroma profile. Li et al. [21] used *Lactobacillus casei* CICC 20995 for the solid-state fermentation of soybean flour. The fermented soybean flour showed improved nutritional value and reduced levels of antinutritional factors. Zhao et al. [22] reported that the fermentation of soy milk with *Lactobacillus delbrueckii subsp. bulgaricus* significantly reduced its beany taste and enhanced its overall aroma. These findings highlight the potential of fermentation as a practical approach to improving the nutritional and sensory quality of legume-based foods.

Kidney beans represent a globally important legume crop with a substantial consumer base, particularly in Latin America, where they constitute a dietary staple [23]. They are also recognized as a valuable source of protein (22.06–32.63%, wet basis), carbohydrates (39–47%, dry basis), and dietary fiber (16.21–24.50%, dry basis) [24]. Moreover, they contain other health-promoting compounds, such as polyphenols, bioactive peptides, and resistant starch [25]. Aydar et al. [26] developed a kidney bean-based beverage; however, the resulting plant milk had a strong beany odor. Given the previously reported benefits of fermentation for flavor improvement, applying LAB fermentation to kidney bean-based milk may represent a promising strategy for improving its nutritional and sensory quality.

In this study, *Lactiplantibacillus plantarum* and *Limosilactobacillus fermentum* were used to ferment white kidney bean-based milk. The nutritional quality of the resulting white kidney bean-based beverage (WKBB) was comprehensively assessed by analyzing pH and viable bacterial counts, physicochemical and nutritional properties, changes in total phenolic and flavonoid contents during in vitro digestion, as well as the bioaccessibility of these compounds. Sensory characteristics and consumer acceptability were also assessed through colorimetric analysis, volatile aroma compound analysis, and sensory evaluation. Our study aimed to develop a nutritionally balanced bean-based beverage with a distinctive flavor and high phenolic bioaccessibility, thereby facilitating the development of novel plant-based food products.

## 2. Materials and Methods

### 2.1. Strain Cultivation

The experimental strains *Lactiplantibacillus plantarum* JCM 1149 and *Limosilactobacillus fermentum* CIP 102980 were non-commercial strains donated by the College of Food Science and Engineering of Northeast Agricultural University (59 Mucai Road, Harbin, China). They were preserved as glycerol stocks in the laboratory of the Faculty of Food Science and Engineering, Kunming University of Science and Technology. Before the experiments, the strains were activated twice. The detailed activation procedure was as follows: the frozen cultures were retrieved from a −80 °C freezer, thawed, and then inoculated into 50 mL of de Man, Rogosa and Sharpe (MRS) broth and incubated at 37 °C for 12 h to obtain the first subculture. Subsequently, 2% (*v*/*v*) of the first subculture was transferred into fresh MRS broth and incubated under the same conditions for another 12 h to complete the activation process. After activation, the bacterial cells were harvested by centrifugation at 815× *g* for 10 min at 4 °C and washed twice with 0.85% (*w*/*v*) sterile saline to remove residual medium. The supernatant was then discarded, and the cell pellet was retained for subsequent inoculation. After activation, the two cultures had the same viable cell concentration and were retained for subsequent inoculation.

### 2.2. Preparation of White Kidney Bean-Based Beverage (WKBB)

White kidney beans were purchased from Yunnan Dianyuan Food (Lijiang) Co., Ltd. (Lijiang City, China). Sugar was purchased from Kunming Songguan Food Co., Ltd. (Kunming, China), and baking soda was purchased from Kunming Ruiyu Food Co., Ltd. (Kunming, China).

Before the experiment, intact and plump white kidney beans without insect damage were selected. The beans were soaked in a 0.25% NaHCO_3_ solution at a bean-to-water ratio of 1:6 (*w*/*v*) for 12 h to promote softening before further processing, rinsed with clean water, and subsequently boiled in water for 10 min to inactivate lipoxygenase, reduce the beany flavor, and decrease lectin activity [27,28]. The treated beans were then ground into a slurry using a blender (PB17001, Guangdong Galanz Microwave Electrical Appliances Manufacturing Co., Ltd., Foshan, China) at a bean-to-water ratio of 1:5 (*w*/*v*) and a rotational speed of 25,000 rpm for 20 min. The resulting plant milk was filtered through a 200-mesh sieve to obtain smooth white kidney bean-based milk. Sugar (8%, *w*/*v*) was then added to the milk, dissolved completely, and mixed thoroughly. The formulated white kidney bean-based milk was dispensed into fermentation bottles at 50 mL per bottle and sterilized at 95 °C for 20 min. After cooling, the white kidney bean-based milk was inoculated with the prepared cell suspensions. The inoculum level was 2% (*v*/*v*) for single-strain fermentation (*L. plantarum* or *L. fermentum*) and 1% (*v*/*v*) for each strain in mixed-strain fermentation, resulting in a total inoculum level of 2% (*v*/*v*). Before inoculation, bacterial cells were harvested by centrifugation at 815× *g* for 10 min at 4 °C and washed twice with 0.85% (*w*/*v*) sterile saline to remove residual medium components. Following inoculation, the initial viable LAB count of each fermentation group was 7.73 ± 0.01 log CFU/mL. The white kidney bean-based milk was fermented at 37 °C until the pH decreased to 4.6 (with reference to the relevant Chinese group standard for plant-based protein beverages), which was considered the fermentation endpoint. The samples fermented with *L. plantarum*, *L. fermentum*, and the mixed culture were designated as LP, LF, and MIX, respectively, while the unfermented control sample was designated as UF. Unless otherwise stated, all samples (UF, LP, LF and MIX) used in the subsequent experiments were fresh liquid samples.

### 2.3. Determination of pH and Viable Bacterial Count

The pH and viable bacterial counts were monitored at 2 h intervals during fermentation. The pH of the samples was measured using a pH meter (PHS-3C, Shanghai Yidian Scientific Instrument Co., Ltd., Shanghai, China). Viable cell counts were determined using the plate count method. Samples (400 μL) were collected from each group every 2 h. They were thoroughly shaken before sampling to ensure complete mixing. The collected samples were then added to test tubes containing 3.6 mL of 0.85% physiological saline and vortexed to ensure thorough mixing. Subsequently, serial dilutions were performed until appropriate dilution factors (10^5^ and 10^6^) were achieved. Next, 100 μL aliquots of the diluted solutions were spread onto MRS agar plates and incubated at 37 °C for 48 h. After incubation, the colonies formed on the plates were counted to determine the viable cell count.

### 2.4. Determination of Titratable Acidity

Titratable acidity, expressed in Thörner degrees (°T), was determined according to the method of Dhakal et al. [29], with minor modifications. In brief, 10 g of the sample was mixed with 20 mL of distilled water. Two drops of phenolphthalein solution were then added as an indicator. The mixture was titrated with 0.1 mol/L sodium hydroxide (NaOH) solution, and the volume of NaOH consumed was recorded. The titratable acidity (°T) was calculated by multiplying the recorded volume of NaOH by 10.

### 2.5. Determination of Soluble Protein Content

Soluble protein content was determined using the Bradford method as described by Meng et al. [30], with minor modifications. In brief, 1 g of the sample was mixed with 40 mL of ultrapure water and centrifuged at 5758× *g* for 10 min. The resulting supernatant was used for the assay. Then, 0.5 mL of the supernatant was mixed with 0.5 mL of ultrapure water and 5 mL of Bradford reagent, vortexed thoroughly, and allowed to stand for 5 min. Following this, the absorbance was measured at 595 nm. Bovine serum albumin was used as the standard.

### 2.6. Determination of Soluble Peptide Content

The extraction method was identical to that used for soluble proteins. Soluble peptides were determined according to the method described by Bai et al. [31], with minor modifications. In brief, 3 mL of the test solution was mixed with an equal volume of 10% trichloroacetic acid and incubated at 37 °C for 10 min. Biuret reagent was then added at a 3:2 (*v*/*v*) ratio, and the reaction was continued at 37 °C for 15 min. The absorbance was measured at 540 nm, with bovine serum albumin used as the standard.

### 2.7. Determination of Total Sugar Content

The total sugar content of the samples before and after fermentation was determined using the phenol–sulfuric acid method, as modified by Zhao et al. [32]. In brief, 1 mL of the sample was diluted with an equal volume of distilled water. Subsequently, 0.5 mL of 6% (*w*/*v*) phenol solution and 2.5 mL of concentrated sulfuric acid were added. The mixture was then incubated in a boiling water bath for 20 min. The absorbance was measured at 490 nm. A standard curve was generated using glucose as the standard to quantify the total sugar content.

### 2.8. Determination of Fat Content

The crude fat content was determined by the Soxhlet extraction method using petroleum ether as the solvent, according to the procedure described by Madjirebaye et al. [33]. After extraction, most of the solvent was removed from the receiving flask by evaporation in a water bath. The flask was subsequently dried to a constant weight in an oven at 105 °C. The fat content was calculated gravimetrically based on the difference between the mass of the receiving flask before and after extraction.

### 2.9. Determination of Phytic Acid Content

The phytic acid content of the samples was quantified using the BC5840 assay kit (Beijing Solarbio Science & Technology Co., Ltd., Beijing, China), in accordance with the manufacturer’s protocol. The assay is based on the enzymatic hydrolysis of phytic acid by phytase to release inorganic phosphorus, which reacts with ammonium molybdate under acidic conditions to form a blue complex exhibiting characteristic absorbance at 660 nm. The phytic acid content was then calculated from the amount of inorganic phosphorus released.

### 2.10. Determination of Total Soluble Solids (TSS) Content

The TSS content was determined using a handheld refractometer and expressed in degrees Brix (°Brix).

### 2.11. Determination of Total Solids Content

To determine the total solids content, the initial wet mass of each sample (g) was recorded. The samples were then dried in an oven at 105 °C to a constant mass. Subsequently, the final dry mass was recorded, and the total solids content was calculated as the ratio of the dry mass to the initial wet mass, expressed as a percentage (%).

### 2.12. Determination of Free Amino Acid Content

The free amino acid content was determined according to Zhai et al. [34]. For sample preparation, 1 mL of the sample was homogenized with an equal volume of 5% (*w*/*v*) sulfosalicylic acid to precipitate proteins. The resulting mixture was then centrifuged (6000× *g*, 10 min), and the supernatant was subsequently filtered through a 0.22 μm membrane to obtain the final test solution. The analysis was conducted on a Biochrom 30+ amino acid analyzer (Biochrom 30+, Biochrom Ltd., Cambridge, UK). Separation was achieved using a Na-type cation exchange resin column (200 mm × 4.6 mm; 8 μm particle size) with a citrate buffer gradient (pH 3.20, 4.25, and 6.45). The analytical conditions were as follows: injection volume, 20 μL; buffer flow rate, 25 mL/h; ninhydrin reaction flow rate, 10 mL/h; reaction cell temperature, 138 °C; and column temperature gradient, 55 °C, 65 °C, and 77 °C. Reference standards for all amino acids were purchased from Sigma.

### 2.13. Determination of Microstructure

Before analysis, the samples were frozen and subsequently lyophilized to complete dryness using a vacuum freeze-dryer (LC-12N-50A, Shanghai Lichen Instrument Technology Co., Ltd., Shanghai, China) [35]. The lyophilized samples were then mounted on stubs using conductive adhesive and sputter-coated with gold. The microstructure of the prepared samples was then examined using a scanning electron microscope (Apreo 2S, Thermo Fisher Scientific Inc., Waltham, MA, USA) at magnifications of 1000× and 5000×.

### 2.14. In Vitro Simulated Digestion of Total Phenols and Flavonoids in WKBB

In vitro digestion of WKBB was performed according to the standardized INFOGEST protocol described by Minekus et al. [36]. Trypsin, pepsin, artificial saliva, artificial gastric juice, and artificial intestinal juice used in the experiment were purchased from Shanghai Yuanye Biotechnology Co., Ltd. (Shanghai, China), while α-amylase was obtained from Beijing Solabio Technology Co., Ltd. (Beijing, China). To avoid sampling during digestion, separate sample tubes were prepared for each digestion endpoint. Samples collected at the gastric endpoint underwent the oral and gastric phases, while those collected at the intestinal endpoint underwent the oral, gastric, and intestinal phases. The simulated digestion began with the oral phase, during which liquid samples (10 g) were mixed with artificial salivary fluid at a 1:1 (*w*/*v*) ratio. The pH of the mixture was adjusted to 7.0, and the mixture was incubated for 2 min at 37 °C with continuous shaking at 180 rpm. Subsequently, for the gastric phase, the oral digestate was combined with artificial gastric fluid at a 1:1 ratio. The pH was then adjusted to 3.0, and the mixture was incubated for 2 h at 37 °C under the same shaking conditions. For the final intestinal phase, the gastric chyme was mixed with artificial intestinal fluid at a 1:1 ratio, and the pH was adjusted to 7.0. The mixture was then incubated for 2 h at 37 °C with continuous shaking. The digestion was performed in three parallel batches, which were terminated after the oral, gastric, and intestinal stages, respectively, in an ice-water bath. Each digestate was then centrifuged at 8158× *g* for 30 min at 4 °C. The supernatants from each digestion endpoint were collected and stored at −20 °C until analysis.

### 2.15. Determination of Total Phenolic Content (TPC) and Total Flavonoid Content (TFC)

The extraction procedure for TPC and TFC determination was performed according to a previous method with slight modifications [5]. Briefly, 6 g of the sample was mixed with 30 mL of 80% methanol and sonicated for 30 min. The mixture was subsequently centrifuged at 4065× *g* for 15 min, and the resulting supernatant was collected for TPC and TFC determination.

TPC was determined using the Folin–Ciocalteu colorimetric method based on the procedure reported by Managa et al. [37], with minor modifications. In brief, 0.5 mL of the test solution was mixed with 2.5 mL of 10% Folin–Ciocalteu reagent. After thorough vortexing, the mixture was incubated for 8 min. Subsequently, 2 mL of 7.5% sodium carbonate solution was added to the mixture. The mixture was then incubated for 60 min in the dark, and the absorbance was measured at 765 nm using a spectrophotometer (UV-6100, Shanghai Mapada Instruments Co., Ltd., Shanghai, China). A standard curve was generated using gallic acid, and TPC was expressed as milligrams of gallic acid equivalents per 100 g of the sample (mg GAE/100 g).

TFC was determined using the aluminum nitrate colorimetric assay, adapted from the method of Meng et al. [30]. The reaction was initiated by mixing 2 mL of the test solution with 0.5 mL of 5% sodium nitrite solution. After a 6 min incubation period, 0.5 mL of 10% aluminum nitrate solution was added, and the mixture was further incubated for 6 min. Subsequently, 4 mL of 4% NaOH solution was added, and the mixture was immediately vortexed. The final reaction mixture was incubated in a water bath at 25 °C for 15 min. The absorbance was then measured at 510 nm using a spectrophotometer. A standard curve was generated using rutin, and TFC was expressed as milligrams of rutin equivalents per 100 g of the sample (mg RE/100 g).

Bioaccessibility was defined as the ratio of the concentration of bioactive compounds released from the food matrix during in vitro digestion to their initial concentration, and was calculated using the following equation [26]:

(1) $$Bioaccessibility\;\left(BI\right)=\frac{C_{d}}{C_{0}}\times 100\%$$

where *C_d_* is the concentration of bioactive compounds after in vitro digestion and *C*_0_ is the initial concentration of bioactive compounds before digestion.

### 2.16. Determination of Color Difference

The color difference in the samples was measured using a colorimeter (CR-400, Konica Minolta Sensing, Inc., Osaka, Japan). The following parameters were recorded: lightness (L*), redness/greenness (a*), and yellowness/blueness (b*). The total color difference (ΔE*) between the samples was then calculated using the following equation [38]:

(2) $$\Delta E=\sqrt{{\left(\Delta L^{*}\right)}^{2}+{\left(\Delta a^{*}\right)}^{2}+{\left(\Delta b^{*}\right)}^{2}}$$

where L* represents lightness, a* represents the red–green coordinate, and b* represents the yellow–blue coordinate.

### 2.17. Determination of Volatile Aroma Compounds

Volatile aroma compounds were analyzed using headspace solid-phase microextraction coupled with gas chromatography–mass spectrometry (HS-SPME-GC–MS; GCMS-QP2010 Ultra, Shimadzu Corporation, Kyoto, Japan), following the method described by Tang et al. [39], with minor modifications. Specifically, 8.0 g of the sample and 5.0 g of NaCl were accurately weighed into a 20 mL headspace vial, followed by the addition of 30 μL of 2-methyl-3-heptanone solution (1.6 mg/L) as the internal standard. Volatile compounds were extracted at 80 °C for 20 min using a preconditioned PDMS/DVB/CAR solid-phase microextraction fiber. After extraction, the fiber was immediately inserted into the GC injection port and thermally desorbed at 250 °C for 5 min. Separation was performed on an HP-5MS capillary column (30 m × 0.25 mm × 0.25 μm; Agilent Technologies, Santa Clara, CA, USA), with ultrapure helium used as the carrier gas. The oven temperature was initially set at 50 °C and held for 5 min, then increased to 200 °C at a rate of 5 °C/min and held for 2 min, and then increased to 230 °C at 10 °C/min and held for 3 min. Electron ionization was performed at 70 eV, and mass spectra were acquired over an *m*/*z* range of 45–550. Volatile compounds were identified by matching their mass spectra against the NIST 20 mass spectral library (similarity index > 80%) and semi-quantitatively determined using the internal standard method [40].

### 2.18. Sensory Evaluation

The sensory evaluation panel consisted of 20 members from the Faculty of Food Science and Engineering at Kunming University of Science and Technology. The panel included 10 women and 10 men, aged 23–30 years.

All panelists possessed basic knowledge of sensory evaluation, had previously participated in sensory evaluations, and were not allergic to white kidney beans. The sensory attributes of color, appearance, taste, smell, and acceptance were evaluated using a nine-point hedonic scale: 1 = dislike extremely, 2 = dislike very much, 3 = dislike moderately, 4 = dislike slightly, 5 = neither like nor dislike, 6 = like slightly, 7 = like moderately, 8 = like very much, and 9 = like extremely [41].

The sensory evaluation procedure was performed according to the method reported by Saint-Eve et al. with some modifications [42]. The detailed procedure was as follows:

(1) Before the sensory evaluation, panelists received a brief training session. The training included the purpose of the experiment, evaluation procedures, evaluation criteria, and the use of the nine-point hedonic scale. Panelists were instructed to evaluate the samples independently based on their personal perceptions and to refrain from communicating with other panelists during the evaluation.
(2) During the sensory evaluation, all samples were coded with random three-digit numbers and presented to panelists in a randomized order to minimize the effects of sample information and presentation sequence. Each sample (20 g) was served in a transparent, colorless cup and kept at 4 °C before evaluation. Before serving, the samples were brought to 10 °C. Panelists rinsed their mouths with mineral water before and after tasting to reduce sensory carry-over effects between samples.
(3) The sensory evaluation was conducted at 10:00 a.m. in a quiet and odor-free room maintained at 25 °C with stable lighting conditions. The panelists evaluated the samples using a nine-point hedonic scale based on color, appearance, taste, smell, and acceptance. During the evaluation, panelists scored the samples according to their personal preferences, and the completed evaluation forms were collected immediately after the evaluation.

### 2.19. Statistical Analysis

All experiments were conducted using three biological replicates, and the results were expressed as the mean ± standard deviation. Statistical analyses were conducted using IBM SPSS Statistics 27 (Version 27.0.1, IBM Corporation, Armonk, NY, USA). Prior to one-way analysis of variance (ANOVA), the normality of the data distribution and homogeneity of variances were assessed using the Shapiro–Wilk test and Levene’s test, respectively. ANOVA was then performed, followed by Duncan’s multiple comparison test to determine significant differences (*p* < 0.05). Graphical representations of the data were generated using Origin 2024 (Version 10.1, OriginLab Corporation, Northampton, MA, USA).

## 3. Results

### 3.1. pH and Viable Bacterial Count

**Figure 1:**
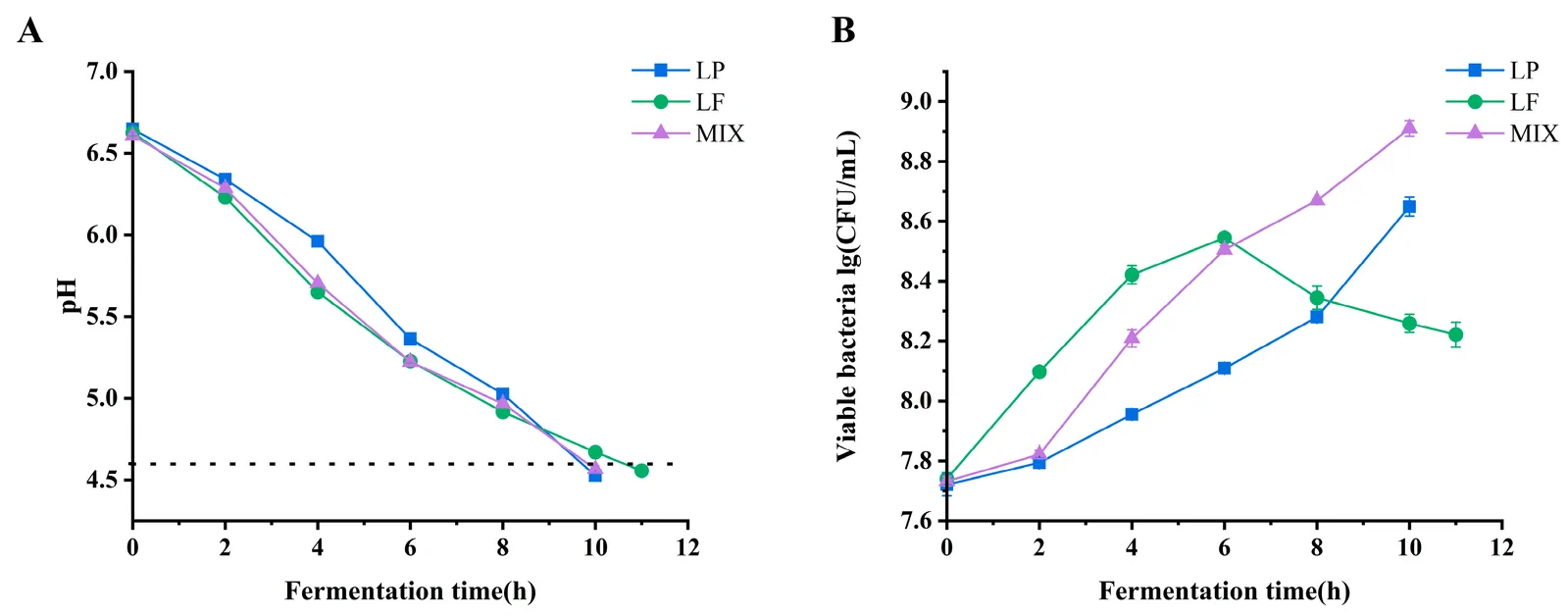
Changes in fermentation parameters of white kidney bean-based beverage. (**A**) pH. (**B**) viable bacteria count. The black dashed line in Figure 1A indicates the predefined fermentation endpoint (pH 4.6). LP: fermentation with *Lactiplantibacillus plantarum*; LF: fermentation with *Limosilactobacillus fermentum*; MIX: co-culture fermentation with both strains.

pH is a key indicator of fermentation. It not only influences the intrinsic characteristics of the final product but also determines product safety and consumer acceptance. Figure 1A illustrates the pH changes during the fermentation of WKBB with different bacterial combinations. The pH of all samples gradually decreased as lactic acid bacteria metabolized sugars to produce lactic acid and other organic acids, resulting in acid accumulation in the medium [43]. This acidification represents a typical biochemical response during lactic acid fermentation, reflecting active microbial metabolism. A similar trend of pH reduction during fermentation was also reported by Sun et al. [20]. A clear difference was noted in fermentation time: the LP and MIX samples reached the fermentation endpoint (pH 4.6) within 10 h, whereas the LF sample required 11 h. These results indicate that the acidification rate of the MIX sample was comparable to that of the *L. plantarum* monoculture and significantly faster than that of the *L. fermentum* monoculture.

The viable bacterial count is a key parameter for assessing the microbial activity of fermented products. It serves as a direct indicator of fermentation stability and the capacity of a product to retain microbial activity during storage. Furthermore, the actual health benefits of a product can be evaluated based on its viable count. Figure 1B presents the changes in viable bacterial counts of the three sample groups during fermentation. The viable counts in all three groups increased during the initial 6 h. Thereafter, the trends diverged: the LP and MIX samples continued to exhibit positive growth, whereas the viable bacterial count in the LF sample began to decline. This decline is likely attributable to acid stress caused by the decreasing pH, which impaired the metabolism of *L. fermentum*, resulting in a death rate that exceeded the growth rate [44]. At the end of fermentation, the viable bacterial counts in all samples reached 8.22–8.91 log CFU/mL, corresponding to approximately 10^8^ CFU/mL. This level is within the viable count range generally considered sufficient for fermented products to provide potential probiotic benefits (>10^6^–10^7^ CFU/mL), suggesting that the fermented WKBB may retain sufficient viable bacteria to exert potential probiotic effects [45]. Interestingly, the similar growth patterns observed in the LP and MIX samples indicate that the presence of *L. plantarum* may contribute to bacterial growth during mixed fermentation. The higher total viable count observed in the MIX sample compared with that of the single-strain fermentation groups may be associated with interactions between the two strains [46]. However, the specific contributions and growth behaviors of each strain in the mixed fermentation system require further investigation using strain-specific identification methods.

### 3.2. Physical and Chemical Properties

The physicochemical parameters of the samples are presented in Table 1. Acidity is a crucial quality parameter in fermented plant-based products. The initial acidity of the UF (unfermented control) sample was 12.00 ± 1.00 °T, and acidity increased in all fermented samples. The MIX sample exhibited the highest acidity, reaching 27.00 ± 1.00 °T. The observed increase in acidity after fermentation is consistent with the results reported by Zhai et al. [34]. The final acidity of a fermented product is determined by the microbial strain and its viable cell count. This is because different strains possess unique metabolic pathways, resulting in variations in the types and production rates of acidic end products [47]. The higher viable bacterial count in WKBB promoted the accumulation of acidic metabolites. Fermentation resulted in a significantly lower soluble protein content than that in the UF sample. The MIX sample exhibited the lowest content at 1.74 ± 0.00 mg/mL, representing a 57.35% reduction from the initial value of 4.08 ± 0.02 mg/mL in the UF sample. In contrast to the soluble protein content, the soluble peptide content was significantly higher in the fermented samples, with the MIX sample showing the greatest increase of 16.03% relative to the UF sample. This inverse relationship may be partly associated with the proteolytic activity of lactic acid bacteria. To acquire essential nitrogen sources for growth, these microorganisms utilize their endogenous proteolytic enzymes to hydrolyze large proteins into smaller, more soluble peptides; this may partly explain the observed decrease in soluble protein content and the concomitant increase in soluble peptide content [48]. A similar decrease in soluble protein content during fermentation was also reported by Bai et al. [31]. They attributed this phenomenon not only to microbial proteolysis but also to protein aggregation and precipitation induced by the decrease in pH during fermentation.

As presented in Table 1, fermentation significantly lowered the total sugar content, with the MIX sample exhibiting the lowest value (74.10 ± 0.38 mg/mL). This reduction may be attributed to the higher viable bacterial count in the MIX sample, as a larger population may produce more α-galactosidase to break down sugars, thereby providing a carbon source for the growth of lactic acid bacteria [43]. Moreover, fermentation significantly reduced the fat content of WKBB (*p* < 0.05). This observation may be attributed to the possible lipolytic activity of lactic acid bacteria; their intracellular lipases may hydrolyze fats into free fatty acids. The liberated free fatty acids, which act as weak acids at pH values above 4.5, may further contribute to the reduction in pH in the final product [49]. Compared with the UF sample, significant reductions in both total soluble solids and total solids were observed in the fermented samples. These decreases may reflect the combined effects of substrate utilization, microbial growth, and metabolite formation during fermentation. A similar decrease in these nutritional components during fermentation was also reported by Bai et al. [31]. They attributed this decrease to the microbial utilization of macromolecular substrates.

Phytic acid is an important antinutritional compound in white kidney beans, and its reduction is essential for improving the nutritional quality of plant-based foods. The phytic acid content of each sample in this study is presented in Table 1. The initial phytic acid content in the UF sample was 7.00 ± 0.13 mg/100 mL. After fermentation, the phytic acid content decreased significantly in all fermented samples, with reductions of 9.86% and 10.71% in the LP and LF samples, respectively, while a greater reduction of 18.43% was observed in the MIX sample. This decrease may be partly associated with the synthesis and secretion of phytase by *L. plantarum* and *L. fermentum* during fermentation [50,51]. These enzymes can catalyze the stepwise hydrolysis of orthophosphate groups from the phytate molecule, thereby generating free inorganic phosphorus and lower inositol phosphates. The removal of these phosphate groups disrupts the complexes that bind phytate to proteins and metal ions. Consequently, nutrient digestibility is enhanced and the antinutritional effects may be mitigated [52]. However, phytase activity was not directly determined in the present study. Therefore, future investigations of the nutritional properties of WKBB should include phytase activity analysis to further verify the role of phytase and clarify its contribution to phytic acid degradation.

### 3.3. Free Amino Acid Content

The contents of 17 free amino acids in the UF, LP, LF, and MIX samples are presented in Table 2. Compared with the UF sample, fermentation lowered the total free amino acid content in all treated samples; this phenomenon has also been reported in previous studies [20,34]. This reduction was particularly pronounced for bitter amino acids, which decreased by 17.56%, 42.21%, and 32.49% in the LP, LF, and MIX samples, respectively. This reduction can likely be attributed to the action of microbial enzymes during fermentation, which catalyze the degradation of amino acids through various processes, such as deamination, transamination, and decarboxylation [53]. Consequently, this process not only mitigates bitterness but also generates volatile compounds (e.g., aldehydes, alcohols, and acids) through amino acid catabolism, thereby enhancing the overall flavor profile of fermented plant-based milk [30].

Based on their nutritional requirements in the human body, amino acids can be classified into EAAs and NEAAs. In this study, the EAA content was quantified to assess nutritional changes. The UF sample contained 67.20 ± 1.17 μg/mL of EAAs. In comparison, the LP sample exhibited a 25.07% lower EAA content, while the LF sample showed no significant change. Notably, the MIX sample exhibited a 14.54% increase in free EAA content. This phenomenon may be related to changes in the amino acid requirements of lactic acid bacteria under mixed-culture conditions [54]. Further analysis indicated that the increase in free TEAA content was primarily attributable to the significant increase in free lysine content, which may be associated with microbial proteolysis during fermentation, as the degradation of protein substrates can promote the release of free amino acids [55]. Lysine is generally limited in plant-based foods, and insufficient lysine intake can lead to decreased immune function, reduced blood protein levels, and impaired physical and cognitive development in children [56]. Therefore, the inclusion of *L. fermentum* CIP 102980 in mixed-strain fermentation may contribute to lysine enrichment and improve the amino acid profile of WKBB.

### 3.4. Microstructural Analysis

**Figure 2:**
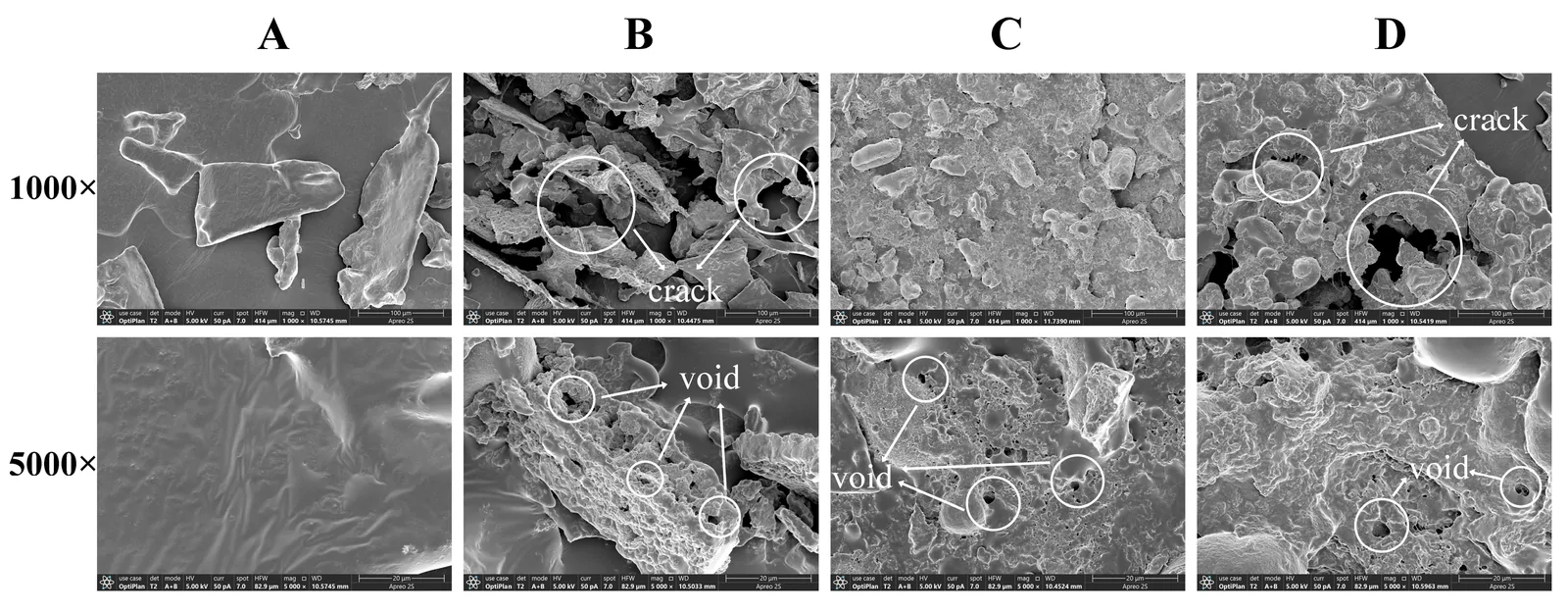
Microstructures of a white kidney bean-based beverage. (**A**) UF: unfermented control; (**B**) LP: fermentation with *Lactiplantibacillus plantarum*; (**C**) LF: fermentation with *Limosilactobacillus fermentum*; and (**D**) MIX: co-culture fermentation with both strains.

The microstructure of each sample group after freeze-drying is presented in Figure 2. The UF sample had a smooth surface with no cracks or voids; no structural fractures were observed, and its fragments were large and intact (Figure 2A). After fermentation with *L. plantarum*, the original structure was markedly disrupted, exhibiting irregular cracks and voids (Figure 2B). At 5000× magnification, the surface appeared uneven and exhibited a distinct network-like porous morphology. This transformation was likely induced by acid production by lactic acid bacteria during fermentation, which caused protein aggregation and structural rearrangement [57]. The microstructure of the plant-based beverage fermented with *L. fermentum* is shown in Figure 2C. The surface of this sample was denser than that of the LP sample, although more protrusions were observed. However, pores with irregular sizes and uneven distribution were only observed at 5000× magnification. This microstructural difference is likely attributable to the adhesive properties of extracellular polysaccharides secreted by *L. fermentum* [58]. These polysaccharides may promote the formation of a relatively dense matrix. The microstructure of the MIX sample is depicted in Figure 2D; this sample exhibited characteristics derived from both single-strain fermentations. At 1000× magnification, the surface exhibited some protrusions, faults, and pores. Despite these surface irregularities, a relatively dense network-like morphology was evident at 5000× magnification. Previous studies have reported that the decrease in pH during fermentation can enhance protein–protein interactions and improve microstructural stability [31].

### 3.5. In Vitro Digestion of Total Phenolics and Flavonoids in WKBB

To further evaluate the impact of fermentation on the nutritional characteristics of WKBB, the changes in total phenolic content (TPC) and total flavonoid content (TFC) in raw white kidney beans, unfermented samples, and fermented samples before and after in vitro digestion were determined. The corresponding results and calculated bioaccessibility values are summarized in Table 3. The raw white kidney beans exhibited TPC and TFC values of 46.23 ± 0.87 mg GAE/100 g and 23.81 ± 0.67 mg RE/100 g, respectively. The TPC and TFC contents of the UF sample were 28.97 ± 0.10 mg GAE/100 g and 7.04 ± 0.18 mg RE/100 g, respectively, whereas those of the fermented samples ranged from 11.60 ± 0.26 to 12.05 ± 0.45 mg GAE/100 g and from 5.50 ± 0.09 to 6.00 ± 0.17 mg RE/100 g, respectively. These results indicate that the TPC and TFC contents of the soaked and boiled beans after blending were higher, although the bean-to-water ratio (1:5) during blending resulted in an approximate fivefold dilution. Similar phenomena have been reported previously, and researchers have attributed this increase to changes in the bean cell wall matrix caused by soaking and boiling treatments, which promoted the release of bound phenolic compounds [59,60]. Meanwhile, all fermented samples showed decreased TPC and TFC contents in the undigested state compared with the UF sample. This decrease may be attributed to the formation of peptides during protein degradation, which may provide potential binding sites for phenolic and flavonoid compounds [61]. The increased concentration of peptides, resulting from proteolytic activity during fermentation, may facilitate the formation of bound complexes with phenolic and flavonoid compounds, thereby lowering the measurable levels of their free forms.

Throughout the simulated digestion process, the TPC and TFC of the UF sample decreased during the gastric phase and subsequently increased during the intestinal phase. In contrast, while the TFC of the fermented samples followed a similar pattern, their TPC decreased significantly only during the gastric phase. During the oral digestion phase, the TPC value of the UF sample showed a significant difference (*p* < 0.05) compared with the undigested sample; however, the magnitude of the change was limited, indicating that short-term oral digestion had a limited effect on the TPC level in the UF sample [62]. Notably, the TPC values of all fermented samples significantly increased during the oral digestion phase. The bioaccessibility of phenolic compounds is influenced by various factors, including the food matrix, chemical forms, and interactions with other components. Fermentation can modify the pH environment and food matrix characteristics of samples [63]. During the oral digestion phase, the addition of simulated saliva adjusted the pH of the fermented samples from an acidic to a neutral environment. This pH transition may affect the interactions between phenolic compounds and matrix components, thereby altering the accessibility and extractability of phenolic compounds and increasing the measured TPC [64]. The decrease in TPC observed in all samples during the gastric digestion phase may be associated with the acidic environment, which promotes (1) the aggregation of polyphenol–protein complexes, thereby increasing particle size and protecting bound polyphenols, and (2) the degradation of unbound phenolic compounds susceptible to low pH. This dual effect of acidic conditions on phenolic stability has been reported in previous studies [65,66]. During the intestinal phase, the neutral environment facilitates the action of trypsin and bile salts, which may help disrupt the protective polyphenol–protein aggregates formed in the stomach. This process may increase the accessibility of phenolic compounds, thereby contributing to the observed increase in bioaccessibility [26,67].

Upon completion of simulated digestion, the bioaccessibility of both total phenolics and flavonoids was significantly higher in the fermented samples than in the UF sample. In particular, the bioaccessibility of total phenolics in the fermented samples ranged from 625.93% to 646.64%, while that of total flavonoids ranged from 43.82% to 48.52%. Tang et al. [61] also reported that the bioaccessibility of total phenolics in yogurt significantly increased after digestion. Thus, fermentation can also be considered an effective approach to enhance the content and bioaccessibility of total phenolics and total flavonoids in WKBB.

### 3.6. Color Difference Analysis

The color of a product directly influences consumers’ purchasing decisions. The colorimetric parameters (L*, a*, b*, and Δ*E*) of all samples are presented in Table 1. Significant differences (*p* < 0.05) were noted among the groups for all measured color parameters. The L* values of all fermented samples significantly increased after fermentation (*p* < 0.05). This is likely attributable to the initial composition of the UF sample, which had higher protein and fat contents. In the UF sample, the particle size of fat globules and protein clusters reduced the L* value by influencing the reflection and scattering of visible light [68]. Furthermore, in our study, the fermentation-induced decrease in pH partially inhibited oxidase activity, resulting in a brighter product color, which is consistent with the findings of Bai et al. [31]. The a* and b* values of the plant-based beverage were also significantly altered after fermentation. The a* values of the LF and MIX samples were significantly higher than those of the UF and LP samples, indicating a shift toward a redder hue. Moreover, the b* values of all fermented samples were significantly higher than those of the UF sample. Such alterations are likely caused by changes in the contents of various components during fermentation, which could modify the light scattering and selective absorption properties of the samples [69]. When the Δ*E* value is > 3, the color change is generally considered noticeable to the naked eye. Compared with the UF sample, all fermented samples exhibited Δ*E* values below 3, indicating that the color changes were generally difficult to distinguish under standardized conditions. Thus, the fermentation process did not result in substantial changes in the appearance of the product.

### 3.7. Volatile Compounds Analysis

**Figure 3:**
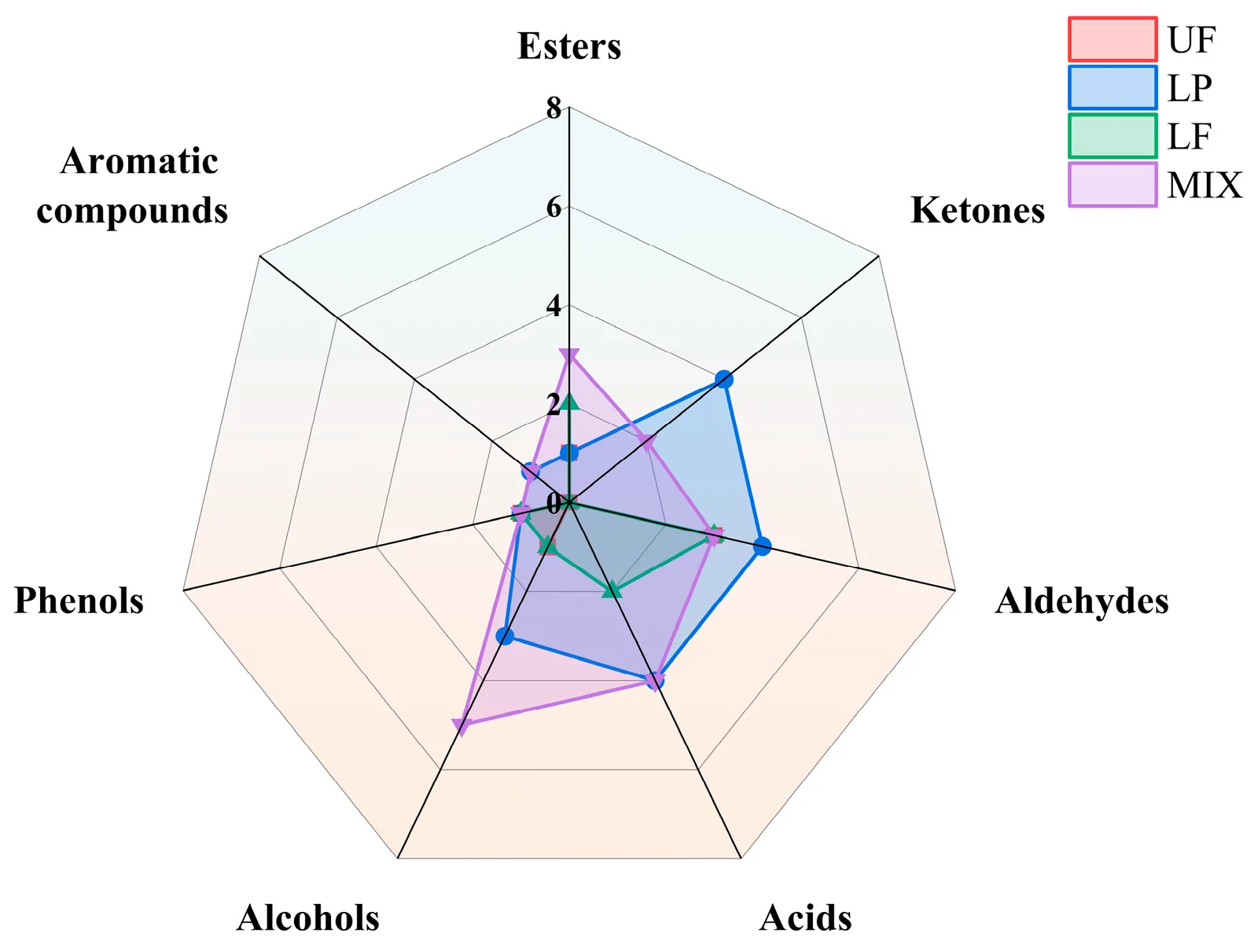
Volatile compounds of white kidney bean-based beverage. UF: unfermented control; LP: fermentation with *Lactiplantibacillus plantarum*; LF: fermentation with *Limosilactobacillus fermentum*; MIX: co-culture fermentation with both strains.

GC–MS analysis identified a total of 25 volatile compounds, which were classified into seven chemical categories: esters (3), ketones (5), aldehydes (4), acids (6), alcohols (5), phenols (1), and aromatic compounds (1). As shown in Figure 3, fermentation markedly increased the overall abundance of volatile compounds in the samples, particularly ketones, acids, and alcohols.

Further analysis of the concentrations of individual volatile compounds is presented in Table 4. Esters are generally formed through esterification reactions between alcohols and organic acids generated during microbial metabolism and are commonly associated with fruity, sweet, and creamy aroma notes [39]. In this study, compared with the UF sample, the total ester contents in the LF and MIX samples increased by 67.14% and 12.20%, respectively, thereby enriching the sensory characteristics of WKBB and imparting a distinctive sweet aroma. However, due to strain-specific characteristics, the total ester content in the LP sample (6.81 ± 0.94 μg/kg) was significantly lower than that of the UF sample. This reduction may be associated with ester transformation during fermentation. Previous studies have suggested that changes in lipase or esterase activities during *Lactobacillus plantarum* fermentation could potentially promote ester hydrolysis [70,71].

Ketones formed during fermentation are primarily derived from the oxidation of saturated fatty acids and the degradation of amino acids [34,72]. In this study, both the diversity and abundance of ketones increased in the LP and MIX samples, thereby enhancing the aroma characteristics of WKBB. Similar findings were reported by Zhai et al. [34], who found that the addition of *Lactiplantibacillus plantarum* significantly increased the concentration of 2-heptanone. Moreover, ketones can exhibit intense aromas even at relatively low concentrations, thereby contributing fruity and sweet notes to the product.

Aldehydes are commonly generated through fatty acid metabolism and amino acid transamination and can impart fruity and floral aroma notes to food [57]. Aldehydes were the predominant volatile compounds in the aroma profile of WKBB; however, their concentrations decreased to varying degrees after fermentation. For example, the concentrations of hexanal and decanal in the MIX sample were 2.40 ± 0.79 and 1.31 ± 0.43 μg/kg, respectively, corresponding to decreases of 11.76% and 41.78% compared with the UF sample. Similarly, the concentration of nonanal in the LF sample was 2.69 ± 0.42 μg/kg, representing a reduction of 7.88% relative to the UF sample. Aldehydes generally have low odor thresholds and may impart pungent or irritating odors at high concentrations. Therefore, the reduction in aldehyde concentrations may contribute to a more pleasant and balanced aroma profile of the product [30].

During fermentation, the strains metabolized available nutrients and produced organic acids, resulting in marked increases in both the abundance and concentrations of acidic compounds. In the present study, the concentrations of acidic compounds increased significantly in all fermented samples (*p* < 0.05). Acetic acid, hexanoic acid, and octanoic acid were the predominant acidic compounds detected. In particular, acetic acid accumulated to 13.14 ± 3.19 μg/kg and 15.16 ± 0.11 μg/kg in the LF and MIX samples, respectively, whereas the concentrations of hexanoic acid and octanoic acid in the LP sample reached 9.86 ± 2.82 μg/kg and 9.73 ± 0.69 μg/kg, respectively. These acidic compounds may contribute cheese-like and floral notes to fermented products; however, their accumulation may also result in an intense sour taste, thereby affecting the sensory characteristics of the products [34]. The sensory effects of acidic compounds depend not only on their individual concentrations but also on their interactions with other volatile compounds. In the MIX sample, the increased contents of alcohols and esters may have contributed to a more balanced aroma profile, thereby reducing the potential negative effects of acidic compounds.

The increase in the content of alcohols is associated with fatty acid reduction, amino acid degradation, and sugar metabolism, among other processes [73]. In addition, aldehydes can be reduced to their corresponding alcohols, which may account for the decrease in aldehyde concentrations and the concomitant accumulation of alcohols observed after fermentation [39]. The total alcohol concentrations in the LP and MIX samples were 8.20 and 11.15 μg/kg, respectively, representing increases of 89.81% and 158.10% compared with the UF sample (4.32 μg/kg). The increase in alcohol content is consistent with the results reported by Zhai et al. [34] and Peng et al. [74]. This phenomenon may be associated with the enhanced utilization of available resources in mixed cultures, where different microorganisms can exploit their complementary metabolic capacities and interact through their metabolites [75].

In addition, 2,4-di-tert-butylphenol was detected in all samples, with significantly higher levels observed in the LF and MIX samples than in the UF sample (*p* < 0.05). This compound is characterized by sweet, citrus-like, and floral aroma notes and may, therefore, make an important contribution to the overall aroma profile [76,77].

Overall, fermentation effectively improved the volatile profile of WKBB by reducing undesirable aldehydes and promoting the formation of desirable aroma compounds, including alcohols, esters, ketones, and organic acids. Notably, the MIX sample exhibited a more balanced and complex aroma profile compared with the aroma profiles of single-strain fermentation samples. These results suggest that mixed-strain fermentation may be an effective strategy for improving the flavor characteristics and sensory quality of plant-based beverages.

### 3.8. Sensory Evaluation of WKBB

**Figure 4:**
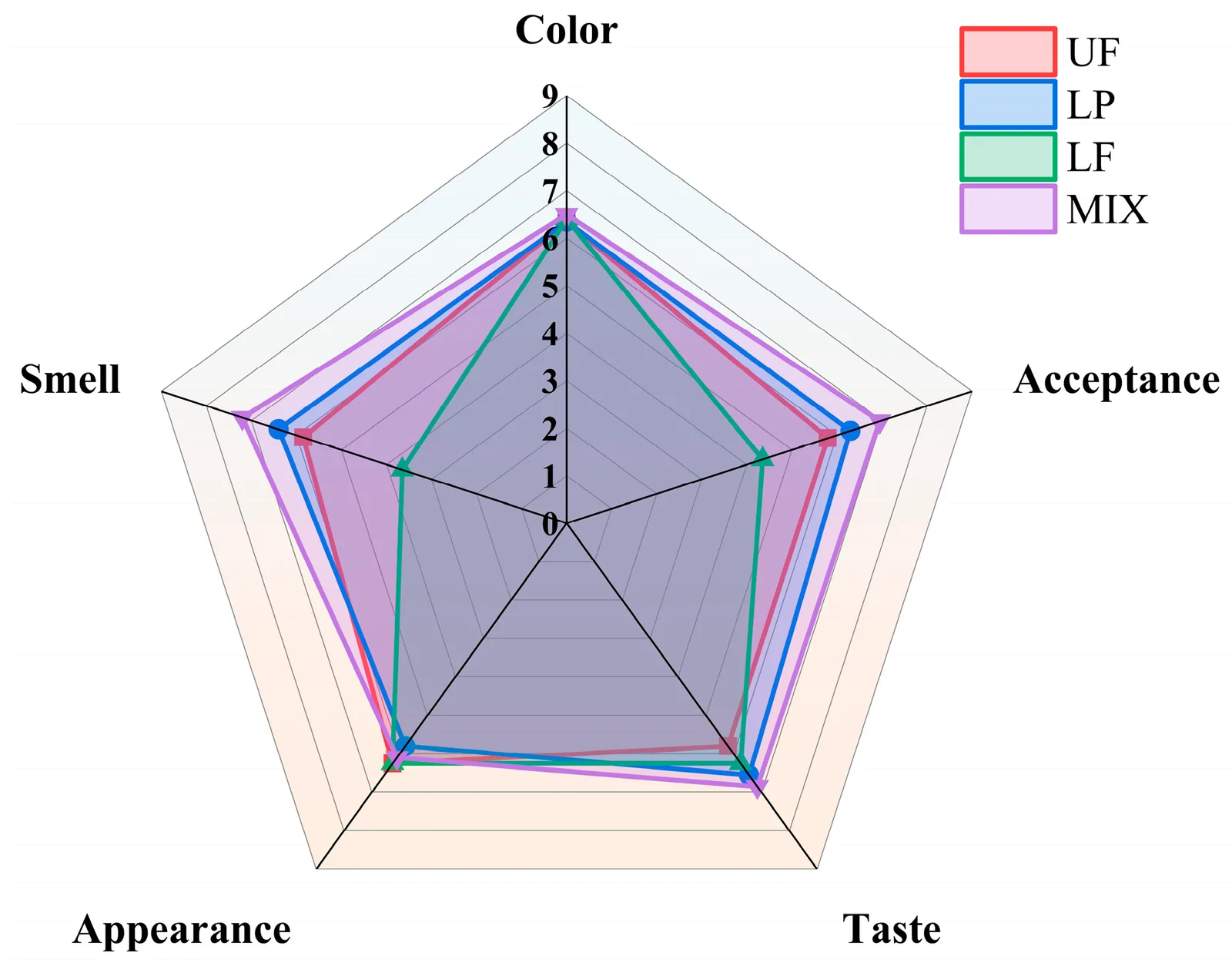
Sensory evaluation of a white kidney bean-based beverage. UF: unfermented control; LP: fermentation with *Lactiplantibacillus plantarum*; LF: fermentation with *Limosilactobacillus fermentum*; MIX: co-culture fermentation with both strains.

The results of the sensory evaluation are presented in Figure 4. These findings align with the instrumental Δ*E* data, which confirmed that fermentation did not induce visually discernible color changes. Regarding aroma, the UF sample was characterized by raw bean and grassy notes, often attributed to certain aldehydes and alcohols [78]. The LF sample also received low scores because of an unpleasant odor, likely resulting from the specific metabolic characteristics of its fermented strain. In contrast, the LP and MIX samples achieved significantly higher aroma scores, which the panel attributed to the absence of undesirable beany odors. The sensory evaluation of aroma showed that the MIX sample received the highest score, followed by the LP and UF samples, while the LF sample exhibited the lowest score. The sensory panel described the UF sample as having a relatively simple aroma profile, mainly characterized by grassy and beany notes, which may be associated with the lack of diverse volatile compounds generated during fermentation. In contrast, the LF sample exhibited more apparent fermented characteristics, with panelists perceiving acidic notes and other indistinct odors. Therefore, the lower aroma score of the LF sample may be related to the balance of its overall volatile composition. Compared with the UF and LF samples, the LP and MIX samples exhibited more diverse aroma characteristics. The sensory panel identified fruity, creamy, and moderately acidic notes in both samples. GC–MS analysis showed that fermentation promoted the formation of various volatile compounds, with increased levels of alcohols, ketones, and some esters observed in the LP and MIX samples. In particular, the MIX sample exhibited a more diverse and balanced volatile profile, which may contribute to greater aroma complexity and improved sensory acceptability of the plant-based beverage. Regarding appearance, the UF sample showed pronounced phase separation and poor homogeneity, whereas the fermented samples exhibited greater visual homogeneity and less apparent phase separation. In the taste assessment, the UF sample scored significantly lower than the fermented samples. The panelists described its flavor as one-dimensional, noting that it lacked the complexity and depth developed during fermentation. The increased acidity in the fermented samples was perceived as a positive attribute, contributing to a more balanced and palatable flavor profile.

Considering these individual attributes, the MIX sample achieved the highest score for overall acceptability.

## 4. Conclusions

The development of novel plant-based foods from underutilized plant resources represents a promising strategy for promoting sustainable food systems. In this study, a white kidney bean-based beverage was developed through lactic acid bacteria fermentation, providing a feasible approach to utilizing white kidney bean resources in novel plant-based food products. Fermentation effectively modified the physicochemical properties, nutritional characteristics, and sensory quality of WKBB, demonstrating its potential to improve the overall acceptability of plant-based beverages. Among the different fermentation strategies, mixed-strain fermentation resulted in a more balanced improvement in product quality, suggesting its potential as a sustainable fermentation approach for the development of white kidney bean-based beverages and other plant-derived foods. However, the present study mainly focused on the quality evaluation and development potential of fermented WKBB, while the underlying mechanisms require further investigation. Future studies should investigate the metabolic mechanisms involved in mixed-strain fermentation, including strain-specific growth interactions, nutrient utilization patterns, and pathways associated with nutritional modification. In addition, further research is required to clarify the mechanisms underlying the formation of volatile compounds during fermentation and optimize fermentation conditions to improve product stability and sensory characteristics. These efforts will provide a deeper understanding of microbial fermentation in plant-based food development and facilitate the practical application of white kidney bean resources in sustainable food production.

## Figures and Tables

**Table 1:** Physical and chemical properties of white kidney bean-based beverage.

Type	UF	LP	LF	MIX
Titratable Acidity (°T)	12.00 ± 1.00 ^c^	25.17 ± 0.76 ^b^	25.00 ± 1.00 ^b^	27.00 ± 1.00 ^a^
Soluble Protein (mg/mL)	4.08 ± 0.02 ^a^	2.03 ± 0.00 ^b^	1.96 ± 0.00 ^c^	1.74 ± 0.00 ^d^
Soluble Peptides (mg/mL)	7.86 ± 0.05 ^d^	8.97 ± 0.05 ^b^	8.76 ± 0.04 ^c^	9.12 ± 0.05 ^a^
Total Sugars (mg/mL)	81.50 ± 0.38 ^a^	76.93 ± 0.76 ^b^	77.58 ± 0.75 ^b^	74.10 ± 0.38 ^c^
Fat (g/100 g)	0.23 ± 0.01 ^a^	0.13 ± 0.02 ^b^	0.08 ± 0.01 ^c^	0.09 ± 0.01 ^c^
Total Soluble Solids (°Brix)	9.77 ± 0.25 ^a^	8.9 ± 0.10 ^b^	8.7 ± 0.20 ^b^	8.8 ± 0.10 ^b^
Total Solids (g/100 g)	11.47 ± 0.07 ^a^	11.18 ± 0.04 ^b^	10.87 ± 0.07 ^d^	11.00 ± 0.07 ^c^
Phytic Acid (mg/100 mL)	7.00 ± 0.13 ^a^	6.31 ± 0.27 ^b^	6.25 ± 0.35 ^b^	5.71 ± 0.23 ^c^
L*	50.20 ± 0.36 ^c^	51.80 ± 0.37 ^b^	52.93 ± 0.28 ^a^	51.77 ± 0.44 ^b^
a*	0.93 ± 0.06 ^c^	1.14 ± 0.06 ^b^	1.19 ± 0.04 ^a^	1.19 ± 0.04 ^a^
b*	7.41 ± 0.06 ^d^	8.02 ± 0.04 ^c^	8.16 ± 0.04 ^b^	8.23 ± 0.06 ^a^
Δ*E*	-	1.74 ± 0.34 ^b^	2.85 ± 0.28 ^a^	1.80 ± 0.40 ^b^

UF: unfermented control; LP: fermentation with *Lactiplantibacillus plantarum*; LF: fermentation with *Limosilactobacillus fermentum*; MIX: co-culture fermentation with both strains. The total color difference (Δ*E*) of the fermented samples was calculated using the UF as the color reference. Results are expressed as mean ± standard deviation. Different letters within the same row indicate significant differences in the data (*p* < 0.05).

**Table 2:** Free amino acid content of white kidney bean-based beverage.

Free Amino Acid	Content (μg/mL)					Taste Attribute
		UF	LP	LF	MIX	
EAA	Threonine	17.65 ± 0.27 ^a^	16.11 ± 0.25 ^b^	15.33 ± 0.35 ^c^	15.68 ± 0.61 ^bc^	Sweet
	Valine	5.48 ± 0.02 ^a^	3.04 ± 0.19 ^c^	2.89 ± 0.06 ^c^	3.44 ± 0.05 ^b^	Bitter
	Methionine	2.70 ± 0.21 ^a^	2.63 ± 0.59 ^a^	2.25 ± 0.48 ^a^	2.45 ± 0.08 ^a^	Bitter
	Isoleucine	6.52 ± 0.13 ^a^	5.21 ± 0.67 ^b^	3.53 ± 0.59 ^c^	3.93 ± 0.82 ^c^	Bitter
	Leucine	2.17 ± 0.19 ^a^	1.79 ± 0.42 ^a^	1.71 ± 0.56 ^a^	1.47 ± 0.10 ^a^	Bitter
	Phenylalanine	17.71 ± 1.08 ^a^	3.27 ± 0.43 ^b^	2.81 ± 0.50 ^bc^	1.89 ± 0.48 ^c^	Bitter
	Histidine	11.01 ± 0.13 ^b^	12.05 ± 0.43 ^a^	8.12 ± 0.35 ^c^	10.51 ± 0.35 ^b^	Bitter
	Lysine	3.96 ± 0.22 ^d^	6.25 ± 0.46 ^c^	28.82 ± 0.46 ^b^	37.61 ± 0.29 ^a^	Bitter
NEAA	Aspartic acid	5.91 ± 0.14 ^a^	3.98 ± 0.07 ^c^	4.14 ± 0.16 ^c^	5.27 ± 0.02 ^b^	Umami
	Serine	23.26 ± 0.43 ^a^	5.74 ± 0.44 ^c^	11.27 ± 0.45 ^b^	5.64 ± 0.22 ^c^	Sweet
	Glutamate	25.50 ± 0.63 ^a^	9.40 ± 0.03 ^c^	9.65 ± 1.22 ^c^	11.07 ± 0.59 ^b^	Umami
	Glycine	2.38 ± 0.02 ^a^	2.19 ± 0.03 ^ab^	1.20 ± 0.09 ^c^	2.00 ± 0.10 ^b^	Sweet
	Alanine	9.97 ± 0.16 ^c^	7.08 ± 0.06 ^d^	27.72 ± 0.09 ^a^	17.53 ± 0.22 ^b^	Sweet
	Cystine	22.96 ± 0.03 ^b^	24.84 ± 1.08 ^a^	23.82 ± 0.61 ^ab^	23.21 ± 0.45 ^b^	Tasteless
	Tyrosine	4.32 ± 0.16 ^a^	3.53 ± 0.21 ^b^	4.29 ± 0.58 ^a^	3.10 ± 0.42 ^b^	Bitter
	Arginine	48.45 ± 1.04 ^a^	46.57 ± 0.53 ^b^	4.72 ± 0.88 ^c^	4.67 ± 0.35 ^c^	Bitter
	Proline	9.41 ± 0.46 ^b^	15.5 ± 0.42 ^a^	7.41 ± 0.15 ^c^	6.89 ± 0.22 ^c^	Sweet
UFAA		31.41 ± 0.48 ^a^	13.38 ± 0.08 ^c^	13.79 ± 1.06 ^c^	16.34 ± 0.62 ^b^	
SFAA		62.66 ± 0.23 ^a^	46.61 ± 0.34 ^c^	62.93 ± 0.14 ^a^	47.74 ± 0.23 ^b^	
BFAA		102.31 ± 0.32 ^a^	84.34 ± 0.99 ^b^	59.13 ± 0.36 ^d^	69.07 ± 1.30 ^c^	
TFAA		22.96 ± 0.02 ^c^	24.84 ± 1.08 ^a^	23.82 ± 0.61 ^ab^	23.21 ± 0.45 ^c^	
TEAA		67.20 ± 1.17 ^b^	50.35 ± 0.90 ^c^	65.45 ± 2.10 ^b^	76.97 ± 0.32 ^a^	
TNEAA		152.15 ± 1.39 ^a^	118.82 ± 1.26 ^b^	94.22 ± 2.47 ^c^	79.38 ± 0.49 ^d^	
Total		219.35 ± 0.40 ^a^	169.17 ± 0.43 ^b^	159.66 ± 0.39 ^c^	156.35 ± 0.60 ^d^	

UF: unfermented control; LP: fermentation with *Lactiplantibacillus plantarum*; LF: fermentation with *Limosilactobacillus fermentum*; MIX: co-culture fermentation with both strains; EAA: essential amino acids; NEAA: non-essential amino acids; TEAA: total essential amino acids; TNEAA: total non-essential amino acids; UFAA: total umami amino acids; SFAA: total sweet amino acids; BFAA: total bitter amino acids; TFAA: total tasteless amino acids. Results are expressed as the mean ± standard deviation. Different letters within the same row indicate significant differences in data (*p* < 0.05).

**Table 3:** In vitro digestion and bioaccessibility of phenols and flavonoids in white kidney bean-based beverage.

	Raw Bean		UF	LP	LF	MIX
TPC (mg GAE/100 g)	46.23 ± 0.87	Non-digested	28.97 ± 0.10 ^aB^	11.60 ± 0.26 ^bD^	11.88 ± 0.34 ^bD^	12.05 ± 0.45 ^bD^
		Oral	28.33 ± 0.17 ^cC^	29.16 ± 0.28 ^bB^	29.61 ± 0.17 ^aB^	29.73 ± 0.23 ^aB^
		Gastric	16.33 ± 0.17 ^cD^	16.82 ± 0.07 ^bC^	16.63 ± 0.30 ^bcC^	17.24 ± 0.06 ^aC^
		Intestinal	70.40 ± 0.49 ^cA^	75.01 ± 0.17 ^abA^	74.36 ± 0.49 ^bA^	75.50 ± 0.12 ^aA^
		BI (%)	243.01	646.64	625.93	626.56
TFC (mg RE/100 g)	23.81 ± 0.67	Non-digested	7.04 ± 0.18 ^aA^	6.00 ± 0.17 ^bA^	5.50 ± 0.09 ^cA^	5.75 ± 0.22 ^bcA^
		Oral	1.62 ± 0.02 ^cC^	1.89 ± 0.03 ^aC^	1.81 ± 0.04 ^bC^	1.90 ± 0.02 ^aC^
		Gastric	0.90 ± 0.03 ^bD^	0.76 ± 0.03 ^cD^	0.71 ± 0.02 ^cD^	0.95 ± 0.02 ^aD^
		Intestinal	2.43 ± 0.01 ^cB^	2.69 ± 0.04 ^bB^	2.41 ± 0.40 ^cB^	2.79 ± 0.02 ^aB^
		BI (%)	34.52	44.83	43.82	48.52

UF: unfermented control; LP: fermentation with *Lactiplantibacillus plantarum*; LF: fermentation with *Limosilactobacillus fermentum*; MIX: co-culture fermentation with both strains; TPC: total phenolic content; TFC: total flavonoid content; and BI: bioaccessibility. Different lowercase letters within the same row and different uppercase letters within the same column indicate significant differences in data (*p* < 0.05).

**Table 4:** Volatile compounds of white kidney bean-based beverage.

Number	Category	CAS	Compound	UF	LP	LF	MIX	Odor Description
1	Esters	141-78-6	Ethyl acetate	16.31 ± 2.14 ^a^	-	10.99 ± 0.86 ^b^	6.96 ± 1.32 ^c^	Fruity, sweet
2		629-33-4	Hexyl formate	-	6.81 ± 0.94 ^c^	16.27 ± 1.09 ^a^	8.32 ± 0.51 ^b^	Fruity, sweet
3		84-74-2	Dibutyl phthalate	-	-	-	3.02 ± 0.18 ^a^	Faint odor
4	Ketones	110-43-0	2-Heptanone	-	5.55 ± 0.32 ^a^	-	1.08 ± 0.21 ^b^	Fruity, spicy, herbal
5		108-94-1	Cyclohexanone	-	-	-	1.98 ± 0.45 ^a^	Minty
6		821-55-6	2-Nonanone	-	5.4 ± 0.60 ^a^	-	-	Fresh sweet, herbal
7		112-12-9	2-Undecanone	-	4.07 ± 1.13 ^a^	-	-	Fruity, creamy
8		593-08-8	2-Tridecanone	-	2.95 ± 0.98 ^a^	-	-	Coconut, nutty
9	Aldehyde	66-25-1	Hexanal	2.72 ± 0.51 ^b^	4.15 ± 0.19 ^a^	2.01 ± 0.07 ^b^	2.40 ± 0.79 ^b^	Fruity
10		124-19-6	Nonanal	2.92 ± 0.44 ^c^	4.54 ± 0.30 ^a^	2.69 ± 0.42 ^c^	3.78 ± 0.18 ^b^	Rose, orange peel
11		112-31-2	Decanal	2.25 ± 0.56 ^ab^	2.86 ± 0.65 ^a^	0.91 ± 0.51 ^c^	1.31 ± 0.43 ^bc^	Sweet, citrus, floral
12		2548-87-0	(E)-2-Octenal	-	0.86 ± 0.32 ^a^	-	-	Fresh cucumber, herbal
13	Acids	64-19-7	Acetic acid	-	-	13.14 ± 3.19 ^a^	15.16 ± 0.11 ^a^	Sour vinegar
14		142-62-1	Hexanoic acid	-	9.86 ± 2.82 ^a^	-	2.70 ± 0.34 ^b^	Sour, cheese
15		124-07-2	Octanoic acid	-	9.73 ± 0.69 ^a^	-	2.51 ± 1.00 ^b^	Vegetable, cheesy
16		97-61-0	Pentanoic acid, 2-methyl-	-	-	0.37 ± 0.08 ^a^	0.29 ± 0.08 ^a^	Sour, cheese
17		112-05-0	Nonanoic acid	-	1.87 ± 0.23 ^a^	-	-	Culture dairy
18		1002-84-2	Pentadecanoic acid	-	8.15 ± 3.73 ^a^	-	-	Waxy
19	Alcohols	513-85-9	2,3-Butanediol	-	-	-	3.17 ± 0.31 ^a^	Fruity, creamy, buttery
20		111-70-6	1-Heptanol	-	-	-	0.83 ± 0.02 ^a^	Herbal
21		3391-86-4	1-Octen-3-ol	-	3.18 ± 0.49 ^a^	-	1.44 ± 0.06 ^b^	Mushroom
22		470-82-6	Eucalyptol	4.32 ± 0.12 ^ab^	2.6 ± 1.32 ^b^	3.94 ± 1.08 ^ab^	5.15 ± 0.61 ^a^	Herbal, camphor
23		111-87-5	1-Octanol	-	2.42 ± 1.35 a	-	0.56 ± 0.24 ^b^	Orange, rose
24	Phenols	96-76-4	2,4-Di-tert-butylphenol	5.84 ± 0.66 ^c^	8.48 ± 2.52 ^c^	12.35 ± 2.69 ^b^	20.11 ± 0.51 ^a^	Phenolic
25	Aromatic compound	118-71-8	Maltol	-	2.68 ± 1.43 ^a^	-	0.99 ± 0.38 ^b^	Sweet, caramel, fruity

UF: unfermented control; LP: fermentation with *Lactiplantibacillus plantarum*; LF: fermentation with *Limosilactobacillus fermentum*; MIX: co-culture fermentation with both strains. “-” means not detected. Different letters within the same row indicate significant differences in data (*p* < 0.05). Odor descriptions were cited from https://thegoodscentscompany.com (accessed on 15 July 2026).

## Data Availability

The original contributions presented in this study are included in the article. Further inquiries can be directed to the corresponding author.

## Abbreviations

The following abbreviations are used in this manuscript:

WKBB	White kidney bean-based beverage
LAB	Lactic acid bacteria
MRS	De Man, Rogosa and Sharpe
UF	Unfermented control
LP	Fermentation with *Lactiplantibacillus plantarum*
LF	Fermentation with *Limosilactobacillus fermentum*
MIX	Co-culture fermentation with *Lactiplantibacillus plantarum* and *Limosilactobacillus fermentum*
TSS	Total soluble solids
TPC	Total phenolic content
TFC	Total flavonoid content
GC-MS	Gas chromatography–mass spectrometry
